# Diversity of CRISPR-Cas immune systems and molecular machines

**DOI:** 10.1186/s13059-015-0816-9

**Published:** 2015-11-09

**Authors:** Rodolphe Barrangou

**Affiliations:** Department of Food, Bioprocessing and Nutrition Sciences, North Carolina State University, Raleigh, NC 27695 USA

## Abstract

Bacterial adaptive immunity hinges on CRISPR-Cas systems that provide DNA-encoded, RNA-mediated targeting of exogenous nucleic acids. A plethora of CRISPR molecular machines occur broadly in prokaryotic genomes, with a diversity of Cas nucleases that can be repurposed for various applications.

## CRISPR-Cas systems and adaptive immunity

**Fig. 1:**
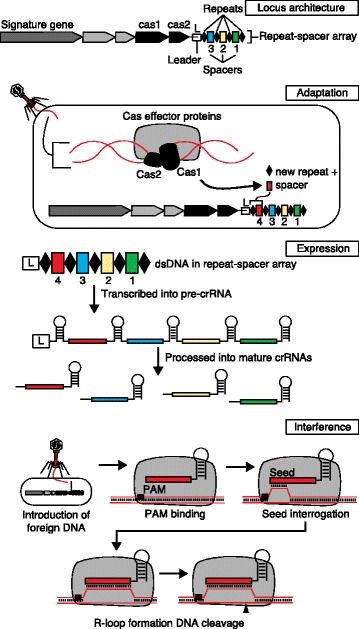
CRISPR-Cas systems and adaptive immunity. CRISPR repeats, together with CRISPR spacers, constitute repeat-spacer arrays that define clustered regularly interspaced short palindromic repeats (CRISPRs). These CRISPR arrays are typically flanked by CRISPR-associated sequences (*cas*) that encode Cas proteins involved in the three stages of CRISPR-encoded immunity, namely adaptation, expression and interference. During adaptation, Cas proteins (including the universal Cas1 and Cas2) sample invasive DNA, leading to the genesis of a new repeat-spacer unit that is inserted in a polarized manner in the CRISPR array. During the second stage — expression — the CRISPR array is transcribed into a full pre-crRNA transcript that is processed into small, mature, interfering CRISPR RNAs (crRNAs). In the third — interference — stage, crRNAs guide Cas effector proteins towards complementary nucleic acids for sequence-specific targeting. Interaction between the interference complex and the target nucleic acid is typically initiated by binding to the protospacer adjacent motif (PAM), which triggers interrogation of flanking DNA by the loaded crRNA. If complementarity extends beyond the seed sequence, an R-loop is formed, and nickase domains within Cas effector proteins cleave the target DNA. *dsDNA* double-stranded DNA, *L* leader

The characterization of biological processes that underlie CRISPR-based adaptive immunity in bacteria and archaea has shaped many crucial aspects of the past decade in the fields of microbiology and genetics, and has enabled the current ‘genome editing’ craze [1]. Clustered regularly interspaced short palindromic repeats (CRISPRs) and their CRISPR-associated (Cas) proteins constitute the CRISPR-Cas immune system (Fig. 1), which provides adaptive immunity against invasive elements such as viruses and plasmids in bacteria and archaea [2–5]. Although CRISPR loci were first observed in the genome of *Escherichia coli* in 1987 [6], it took 15 years of microbial genomics renaissance to appreciate their widespread occurrence in bacteria and archaea [7, 8]. Actually, it was exactly 10 years ago that the first functional clue emerged, with the observation that CRISPR spacers showed homology to viral sequences [9–11], leading to the hypothesis that they might constitute a prokaryotic equivalent to RNA interference (RNAi) [12]. Shortly thereafter, their biological function as adaptive immune systems was established [13], revealing that CRISPR arrays, together with *cas* genes, provide acquired immunity against bacteriophages in a sequence-specific manner. The mechanism of action of various CRISPR-Cas systems has since been determined through milestone discoveries establishing that CRISPR-encoded immunity is mediated by CRISPR RNA (crRNAs) [14], and targets invasive DNA [15] and sometimes RNA [16].

Key discoveries quickly established that targeting is generally dependent upon a short DNA sequence known as the protospacer adjacent motif (PAM) [17–19], is driven by seed sequences [20, 21] and is mediated by Cas endonucleases that specifically cleave complementary DNA [22]. For type I systems, early efforts defined the biochemical and structural underpinning of the ‘CRISPR-associated complex for antiviral defence’ (Cascade) [14], and the endonucleolytic and exonucleolytic degradation of DNA by Cas3 [23–29]. For type II systems, early studies defined crRNA biogenesis [30], Cas9-dependent immunity [13] and cleavage [22], and eventually re-programmable targeting [31] and genesis of precise double-stranded DNA (dsDNA) breaks [32–34].

Arguably, it was the turning of native CRISPR-Cas systems into engineered and programmable two-component systems comprising Cas9 and single guide RNAs (sgRNAs) [33] that was the technological tipping point that singlehandedly enabled Cas9-driven genome editing [35–37] and fuelled the CRISPR craze that has unabatedly unfolded since then [1, 38]. The technical tour de force essentially turned the native Cas9–*trans*-activating CRISPR RNA (tracrRNA)–crRNA–RNase-III four-component system into the streamlined Cas9–sgRNA technology, rendering the challenge of co-opting the system for eukaryotic applications accessible. The synthetic genesis of sgRNAs allowed the repurposing of CRISPR-Cas immune systems into powerful and nimble molecular machines that can yield double-stranded breaks. Indeed, the Cas9 molecular-scalpel-based genome editing craze was foreshadowed in the fall of 2012 [39], following the release of the sgRNA–Cas9 technology, and preceding the publication of proof of concept in human [35, 36] and bacterial cells [37]. Within months, the Church, Zhang and Marraffini labs were able to concurrently establish that the sgRNA–Cas9 technology can be exploited for efficient genome editing, and immediately thereafter, hundreds of studies showed that this approach can be universally implemented in a wide range of cells and model organisms. The avalanche of Cas9-based genome editing studies attests to the potential of this broadly applicable technology.

Mechanistically, CRISPR-Cas immunity hinges on three distinct steps, defined as adaptation, expression and interference (Fig. 1). In the adaptation stage, CRISPR immunization occurs through the uptake and polarized integration of invasive DNA as a novel CRISPR spacer into the CRISPR array, creating a serial record of vaccination events. In the expression stage, the CRISPR array is transcribed into a full pre-CRISPR RNA (pre-crRNA) transcript that is processed into mature crRNAs containing partial CRISPR spacer sequences attached to partial CRISPR repeats, forming CRISPR guide RNAs. In the interference stage, crRNAs guide Cas nucleases towards complementary nucleic acids for sequence-specific targeting and cleavage of invasive genetic elements. Most CRISPR effector proteins initiate targeting by interaction with a particular two-to-four nucleotide sequence motif, the PAM. Once interaction with the PAM has been established, the crRNA guide loaded within the Cas nuclease can then interrogate the flanking target DNA [40, 41]. The strength and duration of the molecular interaction correlates with the level of complementarity between the crRNA and target DNA, which drives conformational changes in Cas effector proteins, such as Cas9 [40, 42, 43] and Cascade [44–46], that eventually lead to a cleavage-competent structural state [40]. If complementarity between the guide RNA and target DNA extends beyond the seed sequence, a DNA R-loop is directionally formed [29, 47, 48], which triggers subsequent nicking by the Cas effector nucleases (i.e., Cas3, Cas9, Cpf1) at particular locations defined by a ruler-anchor mechanism. The literature includes many reviews that cover the history [49–52], biology [3–5, 53–56] and applications [57–63] of CRISPR-Cas systems.

## Diversity of CRISPR-Cas systems and mechanisms of action

**Fig. 2:**
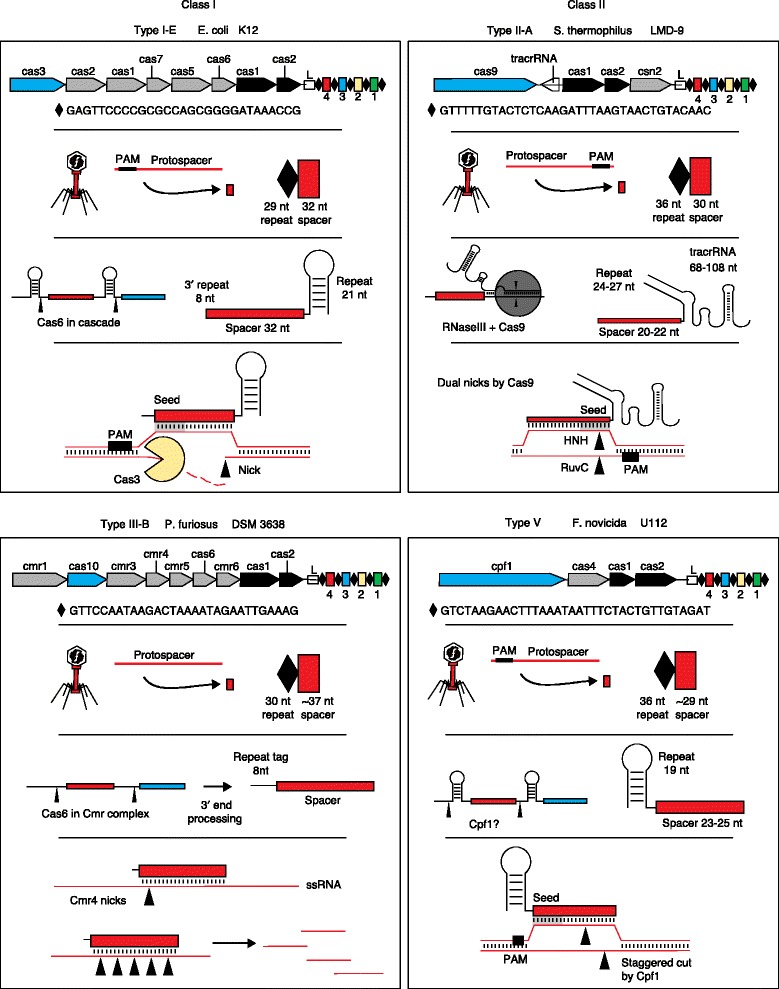
Diversity of CRISPR-Cas molecular machines. Two main classes of CRISPR-Cas systems exist, which are defined by the nature of their Cas effector nucleases, either constituted by multiprotein complexes (class 1), or by a single signature protein (class 2). For class 1 systems, the main types of CRISPR-Cas systems include type I and type III systems. Illustrated here as an example, the *Escherichia coli* K12 type I-E system (*upper left*) targets sequences flanked by a 5′-located PAM. Guide RNAs are generated by Cascade, in a Cas6-defined manner and typically contain an eight-nucleotide 5′ handle derived from the CRISPR repeat, a full spacer sequence, and a 3′ hairpin derived from the CRISPR repeat. Following nicking of the target strand, the 3′ to 5′ Cas3 exonuclease destroys the target DNA in a directional manner. In the *Pyrococcus furiosus* DSM 3638 type III-B system (*lower left*), a short crRNA guide directs the Cmr complex towards complementary single-stranded RNA in a PAM-independent manner. For the canonical type II-A *Streptococcus thermophilus* LMD-9 system (*upper right*), a dual crRNA–tracrRNA guide generated by Cas9 and RNase III targets a 3′-flanked PAM DNA complementary sequence for the genesis of a precise double-stranded break using two nickase domains (RuvC and HNH). For the *Francisella novicida* U112 type V system (*lower right*), a single guide RNA targets complementary dsDNA flanked by a 5′-PAM using Cpf1, which generates a staggered dsDNA break. *Cascade* CRISPR-associated complex for antiviral defense, *CRISPR* clustered regularly interspaced short palindromic repeat, *crRNA* CRISPR RNA, *dsDNA* double-stranded DNA, *L* leader, *nt* nucleotide, *PAM* protospacer adjacent motif, *ssRNA* single-stranded RNA, *tracrRNA* trans-activating CRISPR RNA

In general terms, there are two main classes [64] of CRISPR-Cas systems, which encompass five major types and 16 different subtypes based on *cas* gene content, *cas* operon architecture, Cas protein sequences, and processes that underlie the aforementioned steps (Fig. 1) [65, 66]. The first class is defined by multiprotein effector complexes (Cascade, Cmr, Csm), and encompasses types I, III and IV. In particular, type I systems are the most frequent and widespread systems, which target DNA in a Cascade-driven and PAM-dependent manner, destroying target nucleic acids by using the signature protein Cas3 [26, 28, 67–71] (Fig. 2). Many studies have led to extensive biochemical and structural characterization of the effector proteins and protein–DNA–RNA complexes implicated in type I CRISPR-Cas systems [20, 23, 24, 46, 72–77]. Likewise, type III systems occur frequently in archaea and are characterized by the multiprotein Csm [78–82] or Cmr [16, 83–95] complexes; they operate in a PAM-independent manner and can cleave DNA or RNA by using the signature Cas10 protein together with effector nucleases such as Cmr4 (the RNase within the Cmr complex for type III-B systems) [85, 95] and Csm3 (the RNase within the Csm complex for type III-A systems) [81, 82]. Interestingly, several recent studies have revealed that type III CRISPR-Cas systems can actually target both nucleic acid types, through co-transcriptional RNA and DNA cleavage [80, 82]. Specifically, distinct active sites within the Cas10–Csm ribonucleoprotein effector complex drive co-transcriptional RNA-guided DNA cleavage and RNA cleavage [80]. Type IV systems are rather rare and still remain to be characterized in terms of their distribution and function.

By contrast, the second class is defined by single effector proteins and encompasses types II and V. Type II systems are defined by the popular Cas9 endonuclease [22], which hinges on dual crRNA–tracrRNA guides [30] that direct the RuvC and HNH nickase domains to generate precise blunt DNA breaks in target DNA sequences flanked by a 3ʹ PAM [22, 31–34, 96, 97]. Type V systems are rare, and characterized by the signature Cpf1 nuclease, which is guided by a single crRNA that directs this RuvC-like endonuclease for staggered dsDNA nicking to yield sticky-ends in target DNA sequences flanked by a 5′ PAM [98].

Recently, several studies have shown that, although CRISPR-Cas systems generally function in three distinct stages, involving peculiar molecular processes and various Cas molecular machines, the adaptation and interference steps can actually be coupled [48, 99–101], which is consistent with the priming hypothesis [48, 102–104]. Specifically, differential binding determines whether cognate target DNA should be destroyed as part of the interference pathway, or whether partially complementary sequences should be directed towards the adaptation path [48]. The coupling of the adaptation and interference stages also reflects their co-dependence on Cas9 and PAM sequences in type II systems [100, 101, 105], and implicates a ‘cut-and-paste’ model rather than ‘copy and paste’ [100].

Overall, a broad genetic and functional diversity of CRISPR-Cas immune systems occurs in the genomes of many bacteria and most archaea. Common denominators include DNA-encoded immunity within CRISPR arrays that yield small guide RNAs, which define sequence-specific targets for Cas nucleases and subsequent nucleic acid cleavage. The universal *cas1* and *cas2* genes, implicated in polarized, sequence- and structure-specific integrase-mediated spacer acquisition during the adaptation stage [106–108], are present in all characterized types and subtypes in the two main classes. By contrast, there is substantial variability between classes, types and subtypes concerning the nature, sequence and structure of the CRISPR RNAs and Cas proteins involved, the reliance on and location of PAM sequences, and the nature of the target nucleic acid. Altogether, this illustrates the extensive multi-dimensional diversity of CRISPR-Cas systems, their native biological functions, and the relative potential for various biotechnological and industrial applications.

The diversity of CRISPR-Cas systems reflects their various functional roles. Although the primary established function of CRISPR-Cas systems is adaptive immunity against invasive genetic elements such as plasmids and viruses, several studies have independently implicated them in other functions, including endogenous transcriptional control, as well as resistance to stress, pathogenicity and regulation of biofilm formation [63, 109–114].

Future studies are anticipated to determine the rationale for the distribution biases in various phylogenetic groups, for the absence of CRISPR-Cas systems in so many bacteria, and to unravel the functional links between immunity and other key biological processes such as DNA homeostasis and repair. One intriguing conundrum about CRISPR-Cas systems is their absence in approximately half of the bacterial genomes sequenced to date, despite their intuitive evolutionary value. Another important consideration is whether the observed biases in proto-spacer sampling during adaptation correlate with efficiency biases for the interference stage. Specifically, spacer adaptation biases have been repeatedly observed in type I systems [115, 116] and in type II systems [105, 117], implicating replication-dependent DNA breaks at replication forks, Chi sites and interplay with the RecBCD DNA repair machinery, and so it will be important to determine whether these also explain spacer efficiency variability during interference.

## Applications of native and engineered CRISPR-Cas systems in bacteria

**Fig. 3:**
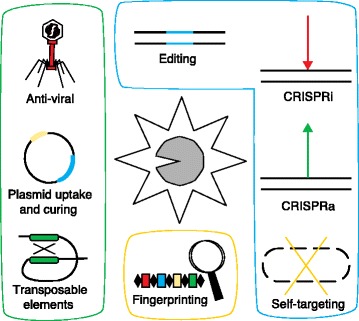
Applications and targets of CRISPR-Cas systems. CRISPR-Cas systems can target various types of nucleic acids, including invasive and mobile DNA (*green box*), or endogenous sequences (*blue box*). In their native environment, CRISPR-Cas systems naturally target mobile and exogenous DNA elements. Conversely, engineered systems are typically designed to target self-DNA to trigger endogenous modifications. Targeting can be directed at bacteriophage DNA to provide anti-viral defense (*upper left*). Likewise, Cas nucleases can be directed at plasmid DNA in order to prevent uptake and dissemination of undesirable sequences or to cure the host of plasmid sequences (*center left*). Targeting can also be directed at mobile DNA elements such as transposons so as to maintain DNA integrity and ensure homeostasis (*lower left*). When aiming the CRISPR-Cas machinery towards the cell’s own chromosomal content, the purpose is typically to induce endogenous DNA repair pathways to drive editing of the DNA sequence (*upper center*). Catalytically deactivated variants of Cas nucleases can be used as DNA-binding proteins to block transcription (CRISPRi, *upper right*), or can be fused to transcriptional activators to activate transcription (CRISPRa, *center right*). Alternatively, Cas nucleases can be reprogrammed to trigger a lethal auto-immune response, leading to cell death (*bottom right*). CRISPR sequences themselves can be used for genotyping, by using the series of vaccination events as a genetic historical record (*lower center*). *Cas* CRISPR associated, *CRISPR* clustered regularly interspaced short palindromic repeat, *CRISPRa* CRISPR activation, *CRISPRi* CRISPR interference

Although the large majority of the CRISPR literature focuses on genome editing applications in eukaryotes, CRISPR-Cas systems arguably afford the most applications in both native and engineered forms in bacteria [118, 119]. Actually, most of the alleged CRISPR literature does not employ bona fide clustered regularly interspaced short palindromic repeats, but instead features crRNA-guided Cas9 proteins. Given the aforementioned CRISPR-Cas system diversity, and the available molecular biology tools for bacteria, we are thus on the cusp of full exploitation in microbes. There are three primary ways to harness CRISPR-Cas systems, depending on the CRISPR immunity stage, Cas machinery and outcome being exploited (Fig. 3).

First, the outcomes of native vaccination events can be used to genotype bacteria by comparing and contrasting the spacer acquisition events to unravel the evolutionary path of a strain isolate, or to delve into assessing the genetic composition and diversity of a population (Fig. 3). This approach has proven valuable for the typing of bacterial pathogens in which CRISPR array diversity reflects functional acquisitions over time, such as in *Escherichia coli*, *Yersinia pestis*, *Mycobacterium tuberculosis*, *Salmonella enterica* and *Clostridium difficile* [120]. This method is also instrumental for the detection and monitoring of industrially valuable workhorses associated with bioprocessing and food manufacturing, such as probiotics and starter cultures [17, 121–124]. Similar approaches, in combination with deep sequencing technology, have shown tremendous potential for the analysis of complex microbial populations, for the determination of clonal population diversity, and for the analysis of co-evolutionary dynamics and arms-races between bacteria and phages [105, 117, 125–127]. Studies can specifically investigate the evolutionary dynamics of hosts and phage populations and unravel selection events and counter-selective mutational patterns that allow bacteria to thrive in inhospitable conditions [128, 129]. In fact, early metagenomic work on CRISPR diversity and interplay with phage sequences from the Banfield laboratory was crucial in establishing the CRISPR field [130, 131]. More recently, studies of CRISPR-based interplay between bacteria and viruses have expanded to human-associated microbial populations, including in the oral cavity and gastrointestinal tract [132–135].

Second, CRISPR-Cas immune systems can be used to vaccinate against invasive genetic elements [13]. Either naturally or by engineering, CRISPR-Cas systems can be exploited to provide resistance against phages [136] or preclude uptake and dissemination of undesirable genetic material such as antibiotic resistance genes [22] or possibly target mobile genetic elements such as transposons to ensure genome homeostasis. In addition to blocking bacteriophages, CRISPR interference is a potent barrier to natural DNA transformation that can be harnessed to prevent the acquisition of virulence traits [137]. Perhaps surprisingly, the industrial exploitation of CRISPR-Cas systems for the genesis of robust and sustainable starter cultures used for the manufacturing of fermented dairy products has been implemented commercially in consumer products for several years [120, 136, 138]. Actually, exploiting first-generation CRISPR patent applications that are over a decade old, and building off early scientific discoveries about adaptive spacer acquisition in *Streptococcus thermophilus*, naturally generated bacteria that have been screened for vaccination events against phage isolated from commercial settings have been exploited on a global scale since 2011. Of course, natural CRISPR-immunized strains might have been used for a long time, unbeknownst to us. Practically, the breadth and depth of phage resistance can be built up iteratively through multiple rounds of selection of natural vaccination events that eventually yield a sustainable starter culture with increased life span in the food industry. Similar approaches hold much potential for the improvement of industrial workhorses valuable to the bio-manufacturing industry.

Third, endogenous or engineered Cas machinery can be repurposed for self-DNA targeting in a wide range of applications that encompass genome editing and targeted killing (Fig. 3). Many studies have documented the nimble potential of the sgRNA–Cas9 technology for ‘traditional’ genome editing, to knock out, insert or delete genes [57–59]. Furthermore, deactivated versions of Cas9 (dCas9) have been generated by inactivation of the RuvC and HNH nickase domains to turn the nuclease into a DNA binding protein able to control transcription, either by blocking RNA polymerases (CRISPR interference, CRISPRi) or by promoting transcription when tethered to transcriptional activators (CRISPR activation, CRISPRa). The use of both endogenous and engineered CRISPR-Cas systems for transcriptional control in bacteria has already been documented [139–141]. More recently, functional variants of Cas9 associated with fluorophores or methylase domains have been used for imaging and epigenome modification [142, 143], respectively. These applications have redefined genome editing beyond the alteration of the DNA sequence per se, and now enable the editing of any sequence in any cell in many ways. Despite the Cas9-based genome editing bias in eukaryotes, their implementation in bacteria is on the rise [118, 144–147]. In bacteria, a promising recent application of self-targeting is programmable killing [148], opening new avenues for the genesis of next-generation smart antimicrobials based on various CRISPR-Cas systems [148–152]. Specifically, engineered Cas9 systems, as well as native Cas9 and Cascade machines, have been successfully re-programmed for sequence-specific targeted killing of a bacterial population, which allows the manipulation of mixed consortia, and the select eradication of defined genotypes of interest [148]. This has successfully been implemented to target *E. coli*, *Streptococcus* and *Staphylococcus aureus*, both in in vitro and in vivo models [148–151]. This is an opportunity to properly select and leverage particular CRISPR-Cas systems that might be better suited for efficient killing, such as type I systems that rely on the Cas3 endo- and exo-nuclease, which digests the target DNA following initial cleavage (Fig. 2), and thus affords the cell fewer opportunities to repair cleaved DNA. Moving forward, there is much potential for this technology to develop narrow-range antibiotics that can be customized for the alteration of microbiomes. This also opens intriguing prospects for programmable eradication of select cell populations in eukaryotes.

**Fig. 4:**
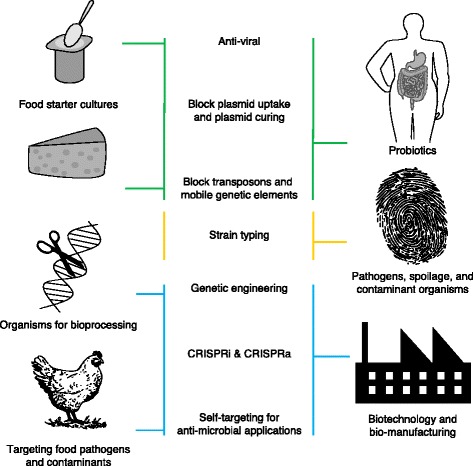
Exploitation of endogenous and engineered CRISPR-Cas systems in bacteria. Exogenous DNA sequences can be targeted by CRISPR-Cas systems to build up phage resistance in food starter cultures (to vaccinate yoghurt strains against bacteriophage), to prevent the uptake and dissemination of plasmids that encode undesirable traits such as antibiotic resistance genes (to immunize probiotic strains used in dietary supplements), or to ensure the genetic integrity and genomic homeostasis of valuable cultures (to fend off mobile genetic elements such as transposons and prophages) (*upper panels*). Unique records of iterative vaccination events captured as a series of spacers in CRISPR arrays can be used as sequencing targets for the detection, monitoring and typing of strains of interest, which include food cultures, spoilage organisms or pathogens (*center panels*). By contrast, self-targeting and engineered applications can be used in industrial settings to improve industrial workhorses by genome editing (indicated by ‘*scissors*’ symbol), or by re-directing the metabolic flux of various pathways for synthetic and yield purposes (*lower panels*). Lethal self-targeting can also be harnessed for the select eradication of pathogens or contaminants of interest. *CRISPRa* CRISPR activation, *CRISPRi* CRISPR interference

Altogether, these various applications illustrate the functional diversity of CRISPR-Cas systems (Fig. 3) and set the stage for the customized selection and development of various molecular machines to expand the molecular biology toolbox. In some ways, type I systems can be construed as a powerful ‘hammer’, which heavily hits and destroys target DNA with the Cas3 exonuclease. Type II systems could be used as nimble ‘screwdrivers’, which precisely target DNA with the Cas9 endonuclease. Similarly, the recently characterized type V systems [64] can be perceived as screwdrivers with a different propensity (flat-head versus phillips) for precise targeting of DNA with the Cpf1 endonuclease [98]. Type III systems can be employed as ‘box cutters’ that can cleave either DNA or RNA with the signature Cas10 nuclease. Given how much our understanding of system diversity has increased in the past 15 years, the diversity of CRISPR-Cas systems will predictably further increase as we deepen our knowledge of microbial genomics, and valuable Cas molecular machines might be unearthed in the future. Altogether, these native and engineered systems hold tremendous potential for a broad range of bacterial applications (Fig. 4).

## Keep calm and CRISPR on

Although the advent of the sgRNA–Cas9 technology for eukaryotic genome editing is merely two years old, the success of this disruptive technology is undeniable [1, 38]. It is noteworthy to point out that the scientific community was primed for the use and rapid implementation of this technology, given the historical use of the powerful RNAi technology on the one hand, and the rise of TALEN-, meganuclease- and zinc-finger nuclease (ZFN)-based genome editing applications on the other. Indeed, many were ready, and well-positioned, if not eager, to unleash the potential of this powerful technology. In hindsight, there are many attributes of CRISPR-Cas systems that render them valuable, including programmability, transferability, efficiency, specificity, affordability, rapidity of implementation, precision, ease of use, and ability to multiplex both guides and systems. Nevertheless, this is still a nascent technology, which needs improvements, especially as it relates to size (Cas9 is arguably cumbersome), targeting flexibility (broadening the PAM space) and efficiency (ability to recognize and cleave targets with specificity and efficiency). Perhaps a longer-term improvement consists of being able to select the most efficient spacer sequences since not all CRISPR spacers or RNA guides provide equal targeting of phage or target sequences, respectively, and adequate prediction of common outcomes (ability of viruses to mutate targeted sequences, or propensities of various DNA repair pathways to alter the cleaved sites). Already, biochemical and structural insights [43, 153–157] are fuelling efforts under way to engineer guides and Cas nucleases for improved functionalities, including smaller variants and PAM-targeting flexibility. In parallel, analysis of Cas nuclease diversity and orthogonality [156, 158–162] will accelerate the rational design of next-generation engineered nucleases. Likewise, lessons from RNAi are instrumental in optimizing the composition and structure of functional CRISPR guides for improved activity and specificity. Finally, the characterization of additional CRISPR-Cas systems in general, and more Cas effector proteins in particular, will broaden the set of forthcoming molecular tools available for various applications.

Already, there are a few valuable lessons regarding Cas effector proteins that have been gathered from CRISPR applications in bacteria that could prove useful to the broad scientific community. In particular, it is noteworthy to point out that, per se, immune systems must afford both specificity and efficiency, so as to prevent auto-immunity and ensure survival, respectively. This is particularly crucial in antiviral CRISPR defence given the speed with which phage co-opt the host cellular machinery, and the ease with which they can mutate to escape sequence-specific targeting. Indeed, stealth and specific targeting of viral DNA occurs through Cas effector protein recognition of bona fide sequences, and their specific nucleolytic destruction. CRISPR-based eradication of phages and toxic DNA thus occurs on the scale of minutes following infection, ensuring efficiency. Likewise, targeting hinging on protospacer recognition ensures that lethal self-targeting events are avoided, providing specificity.

Using recent history and the current momentum to foretell the short-term future of the CRISPR craze, it appears that: first, the pace at which the field is moving forwards is not abating, as indicated by literature output, citation rates and funding trends; second, the coverage has extended feverishly beyond the scientific press, into the mass media; and finally some of the most enthralling level of interest lies in the business commitment and commercial potential of that technology, illustrated by financial investment levels spanning a broad range of business segments, such as medicine, food, agriculture and biotechnology. As the fascinating CRISPR story continues to unfold, and the IP, ethical and awards debates consume attention, it will be crucial to ensure that we *keep calm and CRISPR on* to ensure we do not hinder but, instead, unleash and further advance this powerful technology.

## Abbreviations

Cas: CRISPR-associated sequences
Cascade: CRISPR-associated complex for antiviral defense
CRISPR: clustered, regularly interspaced short palindromic repeats
crRNA: CRISPR RNA
PAM: protospacer adjacent motif
sgRNA: single guide RNA
tracrRNA: trans-activating CRISPR RNA
